# Temporal Modulations of Contact Force during Haptic Surface Exploration

**DOI:** 10.1371/journal.pone.0152897

**Published:** 2016-04-13

**Authors:** Stephanie Mueller, Sven Martin, Michael Schwarz, Martin Grunwald

**Affiliations:** Haptic Research Laboratory, Paul-Flechsig-Institute for Brain Research, Medical Faculty, University of Leipzig, Leipzig, Germany; University of Ottawa, CANADA

## Abstract

Individuals constantly modulate their exploratory movements and adapt their internal hypotheses to incoming sensory information to achieve a thorough and realistic percept. Perception depends on the exploratory movements as well as influencing them. While this seems to be common sense, scientifically we know very little about the temporal dynamics during haptic exploration. To address this, we investigated the exploratory force modulations of two groups of healthy young adults during the exploration of grated surfaces with differing detection difficulty during successive (n = 20) and random stimulus presentation (n = 20). Results showed that exploratory force depended on stimulus properties and increased with increasing detection difficulty. Both experiments yielded the same direction of results with slightly smaller effects in the random stimulus presentation group. Across exploration time average fingertip force also increased. The biggest increase occurred systematically at the beginning (within the first 40 percent) of exploration time per stimulus indicating that most critical information is received during the initial contact phase and is directly transformed into the exploration procedure and force application. Furthermore, video-analyses and comparisons to our high temporal resolution data revealed strong dynamic changes in pressure application during test stimulus exploration with differences in the force dynamics and exploration strategies of simple and difficult stimuli.

## Introduction

The detailed analysis of interactions between sensory and motor information processing is essential for the understanding of haptic perception. Active and goal oriented exploration of objects and surfaces is accompanied by a multitude of physiological, motor and cognitive processes. For these processes to occur it is fundamentally important that a direct physical contact between the organism and the material structure of the object is established. Self-evidently, this active initiation of physical contact is the prerequisite for all subsequently occurring manipulations and control processes. During haptic perception of objects and surfaces, organisms constantly modulate their movements and adapt their internal hypotheses to the incoming sensory information to achieve a thorough and realistic percept. This process is bidirectional, as the perception depends on the exploratory movements as well as it influences them [1–4].

Analogous to Gregory’s perception theory [5], haptic perception may be understood as consisting of sequences of proposing hypotheses and testing them. Therefore, perception may be described as an active constructional process as opposed to passive sensation of environmental stimuli [6]. The perceptual and cognitive success of haptic explorations crucially depends on the adequacy of the dynamic force modulations of the exploring subject. Therefore, the analysis of the associated force characteristics is of central interest for understanding haptic perception.

While variations in force depending on object and surface properties have been reported in various earlier studies [11], the temporal modulations of finger force *during* exploration have (to our knowledge) not been investigated to date. Haptic fingertip force regulation is a broad and highly dynamic process which proceeds spontaneously and extremely variably and is, therefore, difficult to investigate in rigorously controlled experimental settings. Early studies have, however, tried to approach the problem by predefining the fingertip forces the participants were to use in different settings. The results of several experiments indicated that the perceived roughness of grooved surfaces varied with different applied forces (between 1 and 25 oz): the greater the fingertip force of actively exploring participants, the rougher the surfaces were judged [7–9].

To investigate and understand the dynamics of sensory and motor interactions, however, participants have to be free to choose their exploration qualities. An increasing number of studies suggest that participants spontaneously vary their exploration strategies by systematically adapting them to different stimulus properties in order to improve or even to optimize perception [4,10–12].

Individuals who were asked to discriminate between spatial frequencies of grooved surfaces varied their exploratory force depending on the experimental task [13]. The participants significantly increased their fingertip force with increasing groove width [8] and with smaller grain sizes of sandpapers [3]. Furthermore, Tanaka et al. (2012) reported that the participants used greater variations of fingertip force during exploration of sandpapers with smaller grains (smoother surfaces). The authors did not investigate the factors eliciting these variations, however.

In a different experiment, participants searching for small raised or recessed squares on a smooth plane, used significantly higher average fingertip force when exploring for recessed ones. The authors interpreted this phenomenon as an attempt to heighten the probability of stimulus detection by increasing the amount of skin that would penetrate into the squares [14]. Similar adaptivity of haptic force regulation has been reported during grasp and manipulation of objects [15,16]. When exploring the softness of silicon rubber stimuli of varying compliance, participants use stronger forces on hard compared to softer stimuli [10]. During lifting of objects participants increase their grip force depending on the object weight and the resulting load force [17]. Furthermore, grip force was adjusted to variations in load force due to acceleration during movement of grasped objects [18,19]. A similar effect has been reported during artificially induced changes in load force of held objects [20]. At any given load force the grip force also depends on the surface material of objects. More slippery objects elicit consistently higher grip forces [17,21]. Furthermore, grip forces change with age [21,22]. Cole et al. (1999) argue that this may be due to increasing skin slipperiness as well as impaired cutaneous afferent encoding of skin–object frictional properties. Older participants applied significantly greater grip forces when lifting objects with different surface properties than younger adults. Therefore, they adapted their haptic exploration procedures not only depending on variations of a stimulus’ properties but also depending on changes in their personal capabilities and characteristics.

Participants generally show inter-individual differences in their preferred force application. Intra-individually, however, the relative force has been shown to be stable across different settings and tasks [4,8,14,23]. Globally, the mean exploratory force varies depending on the task properties. During evaluation and comparison of stimulus compliance relatively large forces between approximately 7 and 40 N are applied [10,24]. The evaluation of object properties such as roughness [3,13] or friction [25] on the other hand, typically require much lower forces (up to 3N) [10].

Overall, it has been shown that the executed force is adapted to task properties, most likely to optimize perception, and that individuals tend to differ in their preferentially applied force. However, what happens during exploration? What modulations of force occur before the final appropriate force is found? Do modulations occur even after that? And what happens when the detection difficulty of the stimuli is varied? If the perceived roughness increases with contact force [7] relatively smooth stimuli with high detection difficulty might demand stronger fingertip forces and larger variations in force might be expected, similarly to those reported by Tanaka et al [3].

To investigate the following hypotheses the stimuli of the Haptic Threshold Test (*HTT*; Haptik-Labor, Leipzig, [26]) were used. The test consists of 13 test stimuli and one reference stimulus, with grooves and ridges of decreasing distance and, therefore, increasing detection difficulty. All stimuli are covered by an opaque PVC layer to prevent relevant visual input. Two experiments will be conducted to evaluate the possible influence of presentation order on fingertip force.

Our hypotheses are as follows. Based on the findings reported above we expect both exploration time and fingertip force to increase with increasing detection difficulty both during successive and random stimulus presentation. We expect exploration time to increase linearly with detection difficulty (Hypothesis 1) as exploration time has been shown to be linked to stimulus difficulty [27,28]. Fingertip force is expected to show a quadratic expression (Hypothesis 2) due to a limitation in fingertip force that will render perception possible. By means of increasing fingertip force participants might try to squeeze the opaque PVC layer between the grooves and ridges (and consequently the skin of the fingertips). Nevertheless, this procedure will soon reach its painful limits. Keenan et al (2009) measured a maximum voluntary fingertip force of 30N during static pressure. During movement (participants moved their finger up and down along a line) pressure decreased to between 10 and 20N [29]. In previous studies using the Haptic Threshold Test young adults reached haptic thresholds of between test stimulus 9 and 11 [30]. We presume that participants will not increase their fingertip force further after they reached their personal threshold. Therefore, we expect the mean fingertip force to level off, possibly even drop, around test stimulus 10.

Fingertip force has been reported to be primarily influenced by stimulus properties [4,10–12]. If, in our case, the applied force is not influenced by fatigue, pain or expectation effects, we should find similar mean fingertip force values per stimulus for both successive and random presentation. The least pressure should occur during exploration of the reference stimulus due to its very low detection difficulty.

Finally, we expect fingertip force to increase across exploration time per stimulus (Hypothesis 3). Mechanoreceptor responses have been shown to decrease in amplitude during continuous stimulation [31]. Due to these adaptation processes we expect to find a steady increase in fingertip force across the exploration of each stimulus.

Additionally, exploratory results of our high temporal resolution data (milliseconds) of fingertip force modulations will be shown and matched to video data of the exploration process.

To summarize, the objective of the present study is to investigate the modulations of finger force during the exploration of grooved surfaces. We expect that participants will adapt their exploratory fingertip force in accordance with the stimulus properties and their individual perceptual limits. Furthermore, we expect that the participants will use temporal force modulations during the exploration of each stimulus in order to improve their task performance. In the present study, fingertip force, exploration time, and haptic threshold are measured to investigate the force dynamics during active surface exploration.

## Methods

### Participants

Forty healthy adult participants (Experiment A: 10 male, 10 female; aged *M* = 23.0, *SD* = 2.20; Range: 20–27 years; Experiment B: 8 male, 12 female; aged *M* = 23.50, *SD* = 4.29; Range: 19–30 years) took part in the study. In Experiment B data of one female participant had to be excluded from analysis due to a technical error during data acquisition. All participants were right-handed. The participants were naïve to the setting and stimuli. All participants had normal or corrected to normal eyesight. Exclusion criteria (assessed via questionnaire) were neurological and psychiatric disorders, as well as any known polyneuropathy or paresthesia of the hands. Our own pre-studies have shown that especially polyneuropathy caused by liver diseases and paresthesia of the hands of unspecified cause influence the individual haptic threshold measured with the Haptic Threshold Test.

All participants gave written informed consent and were rewarded with University credit points. The study was in accordance with the Declaration of Helsinki and approved by the Medical Ethics Committee of Leipzig University.

### Stimuli

**Fig 1:**
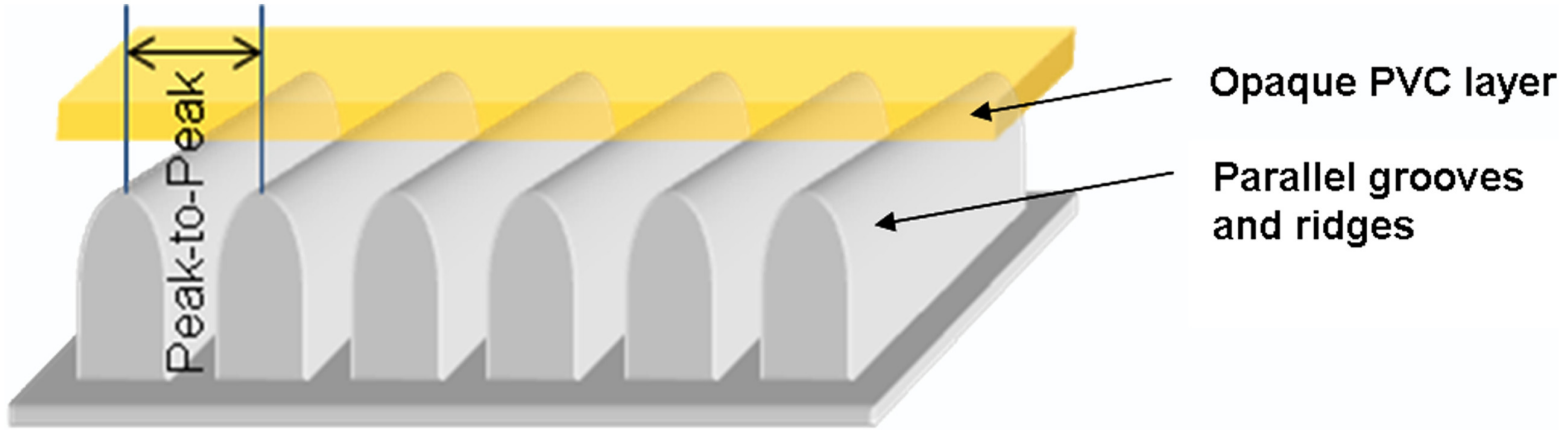
Scheme of the stimulus structure. *Note*. Reproduced from [30] with permission of Springer Science+Business Media.

The stimuli of the Haptic Threshold Test (*HTT*; Haptik-Labor, Leipzig, [26]) were used. Each of the 13 two-dimensional relief stimuli (parallel grooves and ridges) was presented in a small round plastic box that is covered by an opaque PVC layer (Fig 1). With every stimulus, the peak-to-peak distance between the ridges decreased. The resulting palpable deformations ranged from 55 to 2μm (see Table 1). The opaque PVC layer rendered the palpable features invisible allowing the participant’s eyes to remain open at all times. The task of the participants was to explore each stimulus (by rubbing their fingertips back and forth across the PVC layer) and to rotate it to bring the grooves and ridges into a horizontal orientation. Both the test board and the test stimuli are equipped with a magnet. It ensures that the test stimulus is held in place during exploration. It also enables the stimulus to be spun very easily and without force around its middle axis during rotation. All stimuli are designed to be close to the human detection threshold and are therefore very difficult to perceive. When the fingertip is rubbed orthogonally to the horizontal stimulus lines the participants will perceive a slight movement-induced vibration. The further the stimulus is turned away from its horizontal orientation the more the vibration fades. For the more difficult stimuli most participants reported to have had not more than a hunch of perception.

**Table 1:** Technical plastic deformation gauge of the separating PVC layer.

Haptic-Pad Number	Peak-to-Peak value in mm	Elongation value in μm^a^
1	3.0	54.71
2	2.8	45.62
3	2.6	39.40
4	2.4	26.11
5	2.2	25.57
6	2.0	23.42
7	1.8	15.23
8	1.6	12.22
9	1.4	10.69
10	1.2	8.25
11	1.0	7.14
12	0.8	6.48
13	0.6	2.16

^a^Mean elongation values in micrometers of the PVC layer for each test stimulus measured with a force of 150 mN (millinewton) applied by an perpendicular indenter tip.

Reproduced from [30] with permission of Springer Science+Business Media.

For a more detailed description of the HTT see [30].

### Experimental procedure

**Fig 2:**
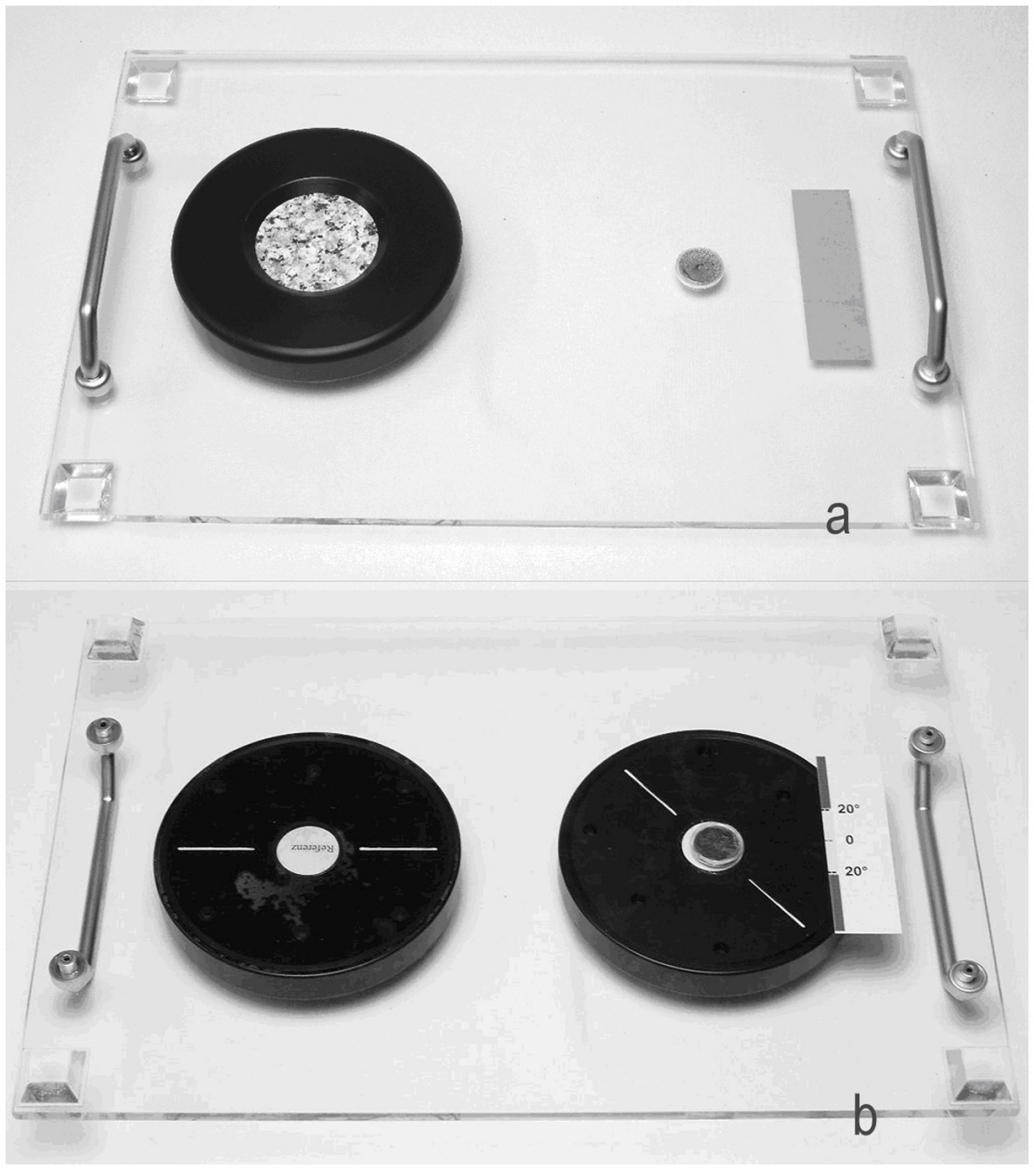
Test board with the reference stimulus on the left side. a) Front view b) Rear view with test stimulus in diagonal orientation (white line) and measurement scale on the right. *Note*. Reproduced from [30] with permission of Springer Science+Business Media.

Participants were seated in a quiet room, on a comfortable chair at a table with the test board in front of them. On the left side of the test board the reference stimulus (peak-to-peak distance 7mm) was fixed in its horizontal orientation (Fig 2a and 2b). Participants were free to explore it at any time as a reference to what a horizontal orientation might feel like. The right side of the test board was used to put the changing test stimuli in a random starting orientation. Before the experiment proper began the setup was explained to the participants with the help of the reference stimulus. To become familiar with the stimuli the participants practiced the task with one easy and one more difficult stimulus (stimuli 1 and 7).

To participants who took part in experiment A the stimuli were presented in ascending order (increasing difficulty). After a short break the more difficult stimuli 8 through 13 were presented a second time to validate the individual threshold. For the statistical analysis of the force dynamics only the data values of the first measurement round will be used. To participants in experiment B all 13 stimuli were presented twice in random order (two test rounds). To enable additional qualitative analyses of the association between movements and fingertip force fingertip movements were videotaped during experiment B.

All participants were free to use any fingers they preferred and to switch between fingers during exploration. The stimulus field had room for up to four fingers or two thumbs. No time limit was given for exploration.

Before the experiment began the finger temperature of each participant was measured with a digital thermometer. In case finger temperature was below 22°C the participants were asked to wash their hands with warm water and rub them try. After a 5 minute acclimatization period finger temperature was measured again (*M* = 27.68, *SD* = 2.80).

To mask any possible sound arising from touching the stimuli the participants wore headphones playing white noise.

The mean experimental exploration time was *M* = 17.52 minutes (*SD* = 8.07) for Experiment A and *M* = 30.06 minutes (*SD* = 20.41) for Experiment B.

### Setup

Two platform scale load cells H10A (Bosch Waegetechnik GmbH & Co.K.G.) of the accuracy class C3 were attached to the test board (one underneath the reference stimulus and one underneath the test stimulus) to measure the applied force during exploration. The measurements were amplified by an instrumentation amplifier (Ultraprecision Operational Amplifier, OP177FP, Analog Devices Inc.). The resulting measurement signals were recorded via the DC voltage channels of an EEG system (EW38, IT-med GmbH, Usingen). Force sensors were calibrated prior to each measurement. Energy for the entire force measurement system was supplied by 12V rechargeble batteries. The exerted fingertip force was measured at a sampling frequency of 256 Hz.

Each stimulus was marked with a white line indicating its horizontal orientation (Fig 2b). Additionally, the test board was marked with a measurement scale. If the white line had an offset of less than 20° the stimulus orientation was considered accurate. To ensure that the examiner could gather and note the offset from horizontal of each test stimulus a camera was installed underneath the test board. For that a camera model RS-OV5116-1330 and a battery-operated monitor (7 inch Nova TFT LCD by X4-Tech) were used.

The fingertip movements during experiment B were recorded directly to computer hard drive using a Sony camera model EVI-D7OP and Debut Video Capture 2.26 (NCH software).

### Statistical Analysis

Force signals were acquired at 256 samples per second with 16-bit resolution, and read using the software Brain Vision Analyzer 2.1 (Brain Products GmbH). SPSS 20 for Windows (IBM Statistics) was used for data manipulation and analysis. Mean and standard deviation for fingertip force per test stimulus and per participant are listed. Also, mean and standard deviation of exploration time per stimulus is given. The association of exploration time and detection difficulty was assessed using Standard Pearson’s correlation. Quadratic regression analyses were performed to analyse the predictive value of detection difficulty on fingertip force. To investigate the possible changes in fingertip force across exploration time per stimulus, the individual exploration time measures (in milliseconds) were converted into percent and then aggregated into 5 exploration time periods (consisting of 20% exploration time each) per participant and test stimulus. The resulting 5 exploration time periods were compared using one-way repeated measures ANOVA and Bonferroni corrected post hoc comparisons.

## Results

**Fig 3:**
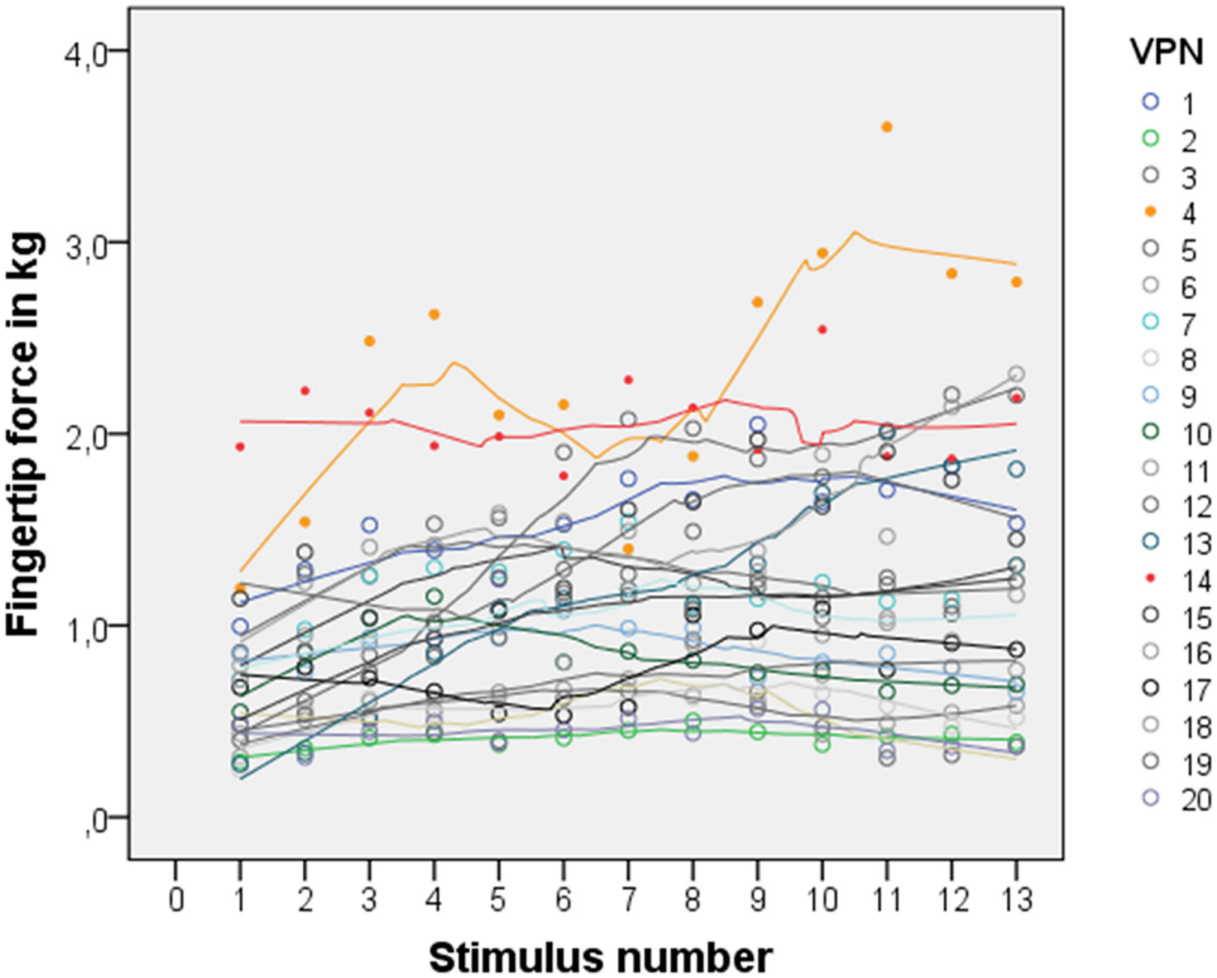
Mean fingertip force of each person for each test stimulus.

The mean haptic threshold reached by the participants of Experiment A was *M* = 9.65 (*SD* = 0.66). During Experiment B the mean haptic threshold was slightly lower with *M* = 8.47 (*SD* = 2.19). The maximum force momentarily applied by a participant was 4.99 kilograms (48.95N; Table 2). With just 1.40 kg (13.74N) participant 3 of Experiment B applied the smallest maximum force. Overall, participants varied greatly in their preferred force. Potentially, as Fig 3 might exemplary indicate, participants may be attributed to two groups: those who increase their fingertip force greatly with increasing detection difficulty and those whose pressure fluctuates around their personal mean. By and large participants maintained their rank position: those who applied more fingertip force on simple stimuli also tended to use greater force for more difficult ones and vice versa. Noticeable were participants 4 and 14 of the first experiment who used especially high fingertip forces from the start and reached mean forces of more than 2 kilograms.

**Table 2:** Descriptive statistics of fingertip pressure per participant during successive (Exp. A) and random (Exp. B) stimulus presentation.

	Experiment A			Experiment B		
Participant Number	*M*	*(SD)*	*Max*	*M*	*(SD)*	*Max*
1	1.41	(0.81)	3.83	1.20	(0.84)	4.81
2	0.43	(0.19)	2.20	1.28	(1.17)	4.99
3	0.54	(0.38)	2.23	0.46	(0.27)	1.40
4	2.52	(1.22)	4.12	-	-	-
5	1.19	(0.83)	3.66	0.68	(0.51)	2.43
6	0.64	(0.27)	1.82	1.35	(1.19)	4.99
7	1.21	(0.72)	3.75	1.73	(1.00)	4.56
8	0.64	(0.37)	2.54	0.54	(0.33)	1.57
9	0.85	(0.39)	1.99	0.94	(0.76)	3.49
10	0.80	(0.48)	2.26	0.95	(0.66)	3.61
11	0.88	(0.49)	2.80	0.81	(0.64)	4.18
12	1.56	(0.81)	3.74	1.03	(0.59)	3.25
13	0.93	(0.77)	3.29	1.19	(0.62)	3.55
14	2.15	(1.01)	4.35	0.59	(0.35)	1.63
15	1.21	(1.32)	4.24	0.46	(0.40)	1.69
16	1.41	(0.68)	3.61	1.25	(1.02)	4.67
17	0.74	(0.51)	2.44	1.43	(1.16)	4.43
18	1.12	(0.57)	3.32	1.12	(0.79)	3.84
19	1.07	(0.64)	3.50	0.89	(0.63)	3.07
20	0.49	(0.29)	1.71	0.56	(0.54)	2.77

Group means of fingertip force did not differ between Experiment A and B (*z* = -1.109, *p* = .267).

### Hypothesis 1

#### Exploration time and detection difficulty

The increasing detection difficulty of the 13 test stimuli was accompanied by a slight increase in mean exploration time per stimulus (*r* = .128, *p* < .05) in Experiment A. On further inspection, test stimulus 13 did not follow in line with the other stimuli (Table 3). Its mean exploration time was as short as the ones of the easiest test stimuli.

**Table 3:** Descriptive statistics of exploration time and fingertip pressure for each test stimulus (Exp. A).

	Exploration time (h:mm:ss)				Fingertip pressure (kg)			
stimulus #	*M*	*(SD)*	*Min*	*Max*	*M*	*(SD)*	*Min*	*Max*
reference	-	-	-	-	0.3871	(0.256)	0.15	1.20
1	0:00:45	(0:00:34)	0:00:14	0:02:49	0.692	(0.406)	0.25	1.93
2	0:00:48	(0:00:21)	0:00:09	0:01:36	0.894	(0.481)	0.31	2.22
3	0:00:54	(0:00:31)	0:00:19	0:02:25	0.997	(0.554)	0.41	2.48
4	0:00:48	(0:00:24)	0:00:16	0:01:34	1.034	(0.554)	0.43	2.62
5	0:00:51	(0:00:24)	0:00:22	0:01:45	1.034	(0.503)	0.38	2.10
6	0:00:52	(0:00:27)	0:00:16	0:01:58	1.105	(0.506)	0.41	2.15
7	0:01:02	(0:00:28)	0:00:16	0:01:49	1.149	(0.532)	0.45	2.28
8	0:00:55	(0:00:29)	0:00:18	0:02:28	1.166	(0.494)	0.44	2.14
9	0:00:59	(0:00:35)	0:00:18	0:02:35	1.214	(0.611)	0.45	2.69
10	0:01:00	(0:00:41)	0:00:19	0:03:09	1.218	(0.704)	0.38	2.94
11	0:01:12	(0:01:00)	0:00:11	0:03:44	1.232	(0.796)	0.31	3.60
12	0:01:05	(0:00:53)	0:00:07	0:03:41	1.199	(0.723)	0.32	2.84
13	0:00:47	(0:00:26)	0:00:05	0:01:47	1.226	(0.727)	0.37	2.79

During random stimulus presentation (Exp. B) the association of detection difficulty and exploration time was somewhat more pronounced (*r* = .310, *p* < .001) with especially long exploration times for stimuli 11 through 13 (Table 4).

**Table 4:** Experiment B: Exploration time per stimulus.

	Round 1				Round 2			
stimulus #	*M*	*(SD)*	*Min*	*Max*	*M*	*(SD)*	*Min*	*Max*
1	0:00:41	(0:00:36)	0:00:11	0:02:15	0:00:40	(0:00:31)	0:00:13	0:02:08
2	0:00:48	(0:00:46)	0:00:10	0:03:07	0:00:40	(0:00:31)	0:00:11	0:01:54
3	0:00:47	(0:00:33)	0:00:10	0:01:48	0:00:53	(0:00:42)	0:00:08	0:02:24
4	0:00:47	(0:00:39)	0:00:13	0:02:18	0:00:43	(0:00:32)	0:00:13	0:02:11
5	0:00:49	(0:00:37)	0:00:10	0:02:35	0:00:53	(0:00:38)	0:00:15	0:02:27
6	0:01:02	(0:00:47)	0:00:16	0:02:47	0:01:02	(0:00:36)	0:00:21	0:02:48
7	0:01:17	(0:00:45)	0:00:18	0:03:11	0:01:08	(0:00:48)	0:00:20	0:02:34
8	0:01:12	(0:00:45)	0:00:17	0:02:58	0:01:09	(0:00:53)	0:00:15	0:03:40
9	0:01:15	(0:00:56)	0:00:18	0:03:41	0:01:12	(0:00:50)	0:00:22	0:03:25
10	0:01:25	(0:01:06)	0:00:34	0:04:22	0:01:19	(0:00:53)	0:00:18	0:03:24
11	0:01:32	(0:01:06)	0:00:29	0:04:31	0:02:05	(0:03:17)	0:00:31	0:15:19
12	0:01:30	(0:01:09)	0:00:13	0:04:45	0:01:40	(0:01:28)	0:00:22	0:06:36
13	0:01:50	(0:01:30)	0:00:26	0:05:40	0:01:35	(0:01:24)	0:00:27	0:05:55

### Hypothesis 2

#### Fingertip force and detection difficulty

**Fig 4:**
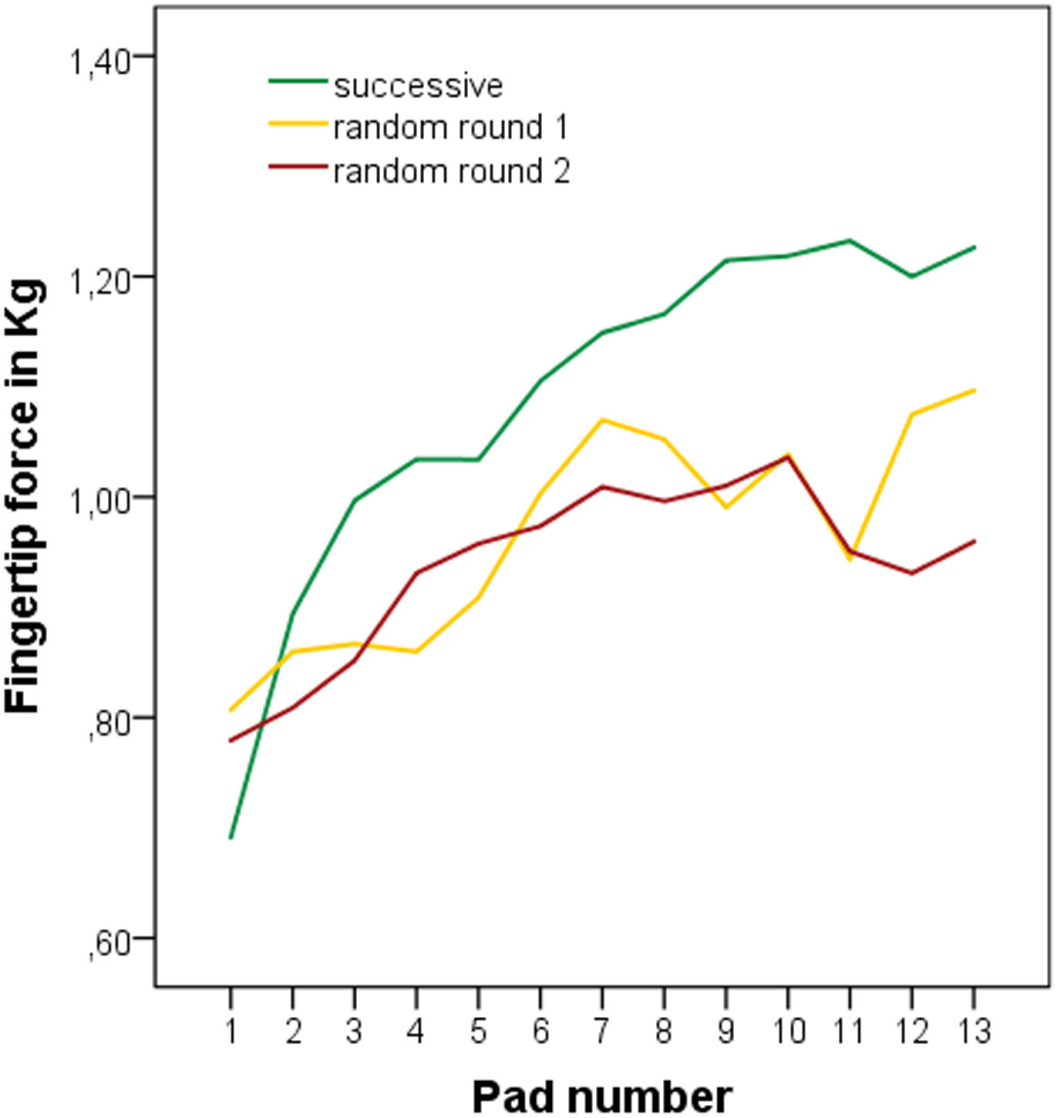
Change in fingertip pressure with increasing detection difficulty.

*Experiment A*: Detection difficulty significantly predicted fingertip force. With increasing detection difficulty two contrasting effects occurred (β1 = 0.104, β2 = -0.005, *p* < .0001, Fig 4). One of these effects caused an increase in fingertip force by 104 grams when detection difficulty is increased by one test stimulus. This linear effect is antagonized by a weak quadratic negative effect.

During low detection difficulty the linear positive effect prevails resulting in increasing fingertip force with increasing difficulty. On average fingertip force increased by 0.5 kilograms from the easiest stimulus to the statistical reversal point (Table 3). In contrast, during high detection difficulty the relationship changed in such a way that fingertip force on average remained stable during exploration of the more difficult stimuli.

However, due to the extensive variance in fingertip force between the participants across all 13 stimuli, the explained variance was small while still highly significant (*R²* = .062, *F* (1,257) = 8.49; *p* < .0001).

*Experiment B*: As in experiment A, with increasing detection difficulty two contrasting effects occurred (β1 = 0.059, β2 = -0.003, *R²* = .036, *F* (2,491) = 9.23, *p* < .005, Fig 4, Table 5). During random stimulus presentation the effects were small but still highly significant in both test rounds (round 1: β1 = 0.047, β2 = -0.002, *R²* = .044, *F* (2,244) = 5.66, *p* < .005; round 2: β1 = 0.071, β2 = -0.004, *R²* = .033, *F* (2,244) = 4.10, *p* < .05).

**Table 5:** Experiment B: Fingertip pressure in kilograms per stimulus.

	Round 1				Round 2			
stimulus #	*M*	*(SD)*	*Min*	*Max*	*M*	*(SD)*	*Min*	*Max*
reference	.09	(.16)	.01	1.10	.09	(.16)	.01	.54
1	.79	(.39)	.24	1.54	.77	(.38)	.33	1.91
2	.83	(.33)	.35	1.47	.78	(.31)	.20	1.29
3	.85	(.31)	.38	1.44	.83	(.34)	.30	1.43
4	.83	(.35)	.32	1.49	.92	(.39)	.36	2.03
5	.89	(.44)	.31	1.89	.94	(.45)	.36	1.99
6	.99	(.48)	.41	2.04	.97	(.42)	.36	1.97
7	1.07	(.49)	.36	2.22	.99	(.46)	.40	1.93
8	1.06	(.48)	.40	1.97	.98	(.42)	.47	2.21
9	.98	(.48)	.37	2.25	1.02	(.51)	.45	2.47
10	1.04	(.47)	.41	1.92	1.03	(.49)	.42	2.08
11	.95	(.47)	.34	2.20	.94	(.50)	.26	2.29
12	1.08	(.45)	.43	2.01	.94	(.41)	.32	2.01
13	1.11	(.58)	.43	2.54	.97	(.45)	.42	2.18

### Hypothesis 3

#### Temporal dynamics of exploratory force

To investigate the possible changes in fingertip force across exploration time per test stimulus during both experiments, the individual exploration time measures (in milliseconds) were converted into percent and then aggregated into 5 exploration time periods (consisting of 20% exploration time each) per participant and test stimulus. For most participants each 20% period consisted of approximately 5 to 20 seconds real time, since all but two participants used roughly 1 minute to explore each stimulus (cf. Table 3).

Descriptive statistics display both increasing mean fingertip forces and again strong interindividual variance of the applied pressure within each of the exploration time periods (Tables 6 and 7).

**Table 6:** Mean fingertip pressure and standard deviation of each test stimulus for each 20% exploration time period during successive presentation (Exp. A).

	20%		40%		60%		80%		100%	
Stimulus #	*M*	*(SD)*	*M*	*(SD)*	*M*	*(SD)*	*M*	*(SD)*	*M*	*(SD)*
1	0.49	(0.28)	0.67	(0.47)	0.68	(0.46)	0.78	(0.51)	0.83	(0.54)
2	0.71	(0.38)	0.85	(0.52)	0.96	(0.52)	0.99	(0.65)	0.96	(0.54)
3	0.75	(0.32)	0.94	(0.64)	1.05	(0.64)	1.09	(0.73)	1.14	(0.78)
4	0.83	(0.42)	1.01	(0.65)	1.11	(0.65)	1.16	(0.72)	1.06	(0.67)
5	0.89	(0.37)	1.03	(0.55)	1.07	(0.55)	1.08	(0.58)	1.08	(0.61)
6	0.97	(0.39)	1.10	(0.60)	1.11	(0.60)	1.19	(0.61)	1.15	(0.66)
7	1.06	(0.47)	1.12	(0.57)	1.16	(0.57)	1.22	(0.63)	1.19	(0.56)
8	1.05	(0.41)	1.23	(0.58)	1.16	(0.58)	1.21	(0.52)	1.18	(0.51)
9	1.13	(0.51)	1.15	(0.70)	1.25	(0.70)	1.28	(0.72)	1.26	(0.69)
10	1.10	(0.58)	1.26	(0.79)	1.31	(0.79)	1.30	(0.86)	1.13	(0.68)
11	1.15	(0.70)	1.28	(0.84)	1.28	(0.84)	1.26	(0.90)	1.19	(0.86)
12	1.04	(0.69)	1.21	(0.83)	1.23	(0.83)	1.27	(0.81)	1.21	(0.69)
13	1.01	(0.54)	1.26	(0.85)	1.22	(0.85)	1.36	(0.91)	1.25	(0.77)
Total	0.94	(0.51)	1.11	(0.64)	1.15	(0.70)	1.19	(0.72)	1.16	(0.69)

**Table 7:** Mean fingertip pressure and standard deviation of each test stimulus for each 20% exploration time period during random presentation (Exp. B).

	1^st^ round										2^nd^ round									
Stimulus	20%		40%		60%		80%		100%		20%		40%		60%		80%		100%	
#	*M*	*(SD)*	*M*	*(SD)*	*M*	*(SD)*	*M*	*(SD)*	*M*	*(SD)*	*M*	*(SD)*	*M*	*(SD)*	*M*	*(SD)*	*M*	*(SD)*	*M*	*(SD)*
1	,84	(,47)	,94	(,47)	,97	(,48)	,93	(,53)	,97	(,53)	,84	(,42)	,99	(,41)	1,02	(,52)	1,03	(,76)	,85	(,44)
2	,89	(,49)	,97	(,37)	1,04	(,54)	1,20	(,55)	,97	(,61)	,94	(,48)	1,06	(,54)	,96	(,40)	1,09	(,53)	1,06	(,53)
3	,86	(,45)	1,01	(,45)	,98	(,36)	1,19	(,57)	1,20	(,53)	,87	(,51)	1,02	(,55)	1,09	(,58)	1,04	(,48)	1,02	(,48)
4	,80	(,51)	1,05	(,51)	1,03	(,53)	1,09	(,54)	1,03	(,61)	,94	(,37)	1,11	(,46)	1,20	(,51)	1,20	(,56)	1,08	(,55)
5	,95	(,58)	1,04	(,62)	1,06	(,53)	1,19	(,75)	1,08	(,55)	,96	(,44)	1,16	(,63)	1,14	(,63)	1,07	(,58)	1,07	(,58)
6	,96	(,53)	1,16	(,61)	1,12	(,73)	1,33	(,66)	1,22	(,57)	,98	(,41)	1,09	(,49)	1,11	(,51)	1,16	(,57)	1,20	(,60)
7	1,03	(,56)	1,19	(,65)	1,21	(,50)	1,24	(,54)	1,21	(,59)	,93	(,40)	1,13	(,58)	1,24	(,76)	1,18	(,66)	1,17	(,60)
8	1,16	(,66)	1,12	(,43)	1,26	(,73)	1,18	(,49)	1,22	(,51)	1,00	(,50)	1,13	(,56)	1,13	(,54)	1,25	(,56)	1,24	(,64)
9	1,07	(,56)	1,14	(,61)	1,15	(,50)	1,19	(,56)	1,08	(,56)	,99	(,52)	1,14	(,55)	1,13	(,76)	1,21	(,65)	1,16	(,58)
10	1,03	(,55)	1,19	(,64)	1,14	(,48)	1,15	(,45)	1,12	(,56)	1,01	(,48)	1,17	(,63)	1,17	(,65)	1,23	(,64)	1,17	(,59)
11	1,00	(,54)	1,07	(,58)	1,00	(,51)	1,13	(,61)	1,15	(,65)	1,01	(,52)	1,06	(,61)	,99	(,57)	1,14	(,64)	1,19	(,61)
12	1,11	(,64)	1,20	(,55)	1,19	(,51)	1,33	(,63)	1,30	(,64)	,98	(,49)	1,12	(,61)	1,09	(,66)	1,09	(,58)	1,11	(,57)
13	1,16	(,61)	1,17	(,61)	1,18	(,68)	1,24	(,73)	1,23	(,67)	1,08	(,53)	1,11	(,47)	1,15	(,58)	1,29	(,74)	1,25	(,67)
Total	,99	(,55)	1,10	(,55)	1,10	(,55)	1,18	(,59)	1,14	(,58)	,96	(,46)	1,10	(,54)	1,11	(,59)	1,15	(,61)	1,12	(,57)

**Fig 5:**
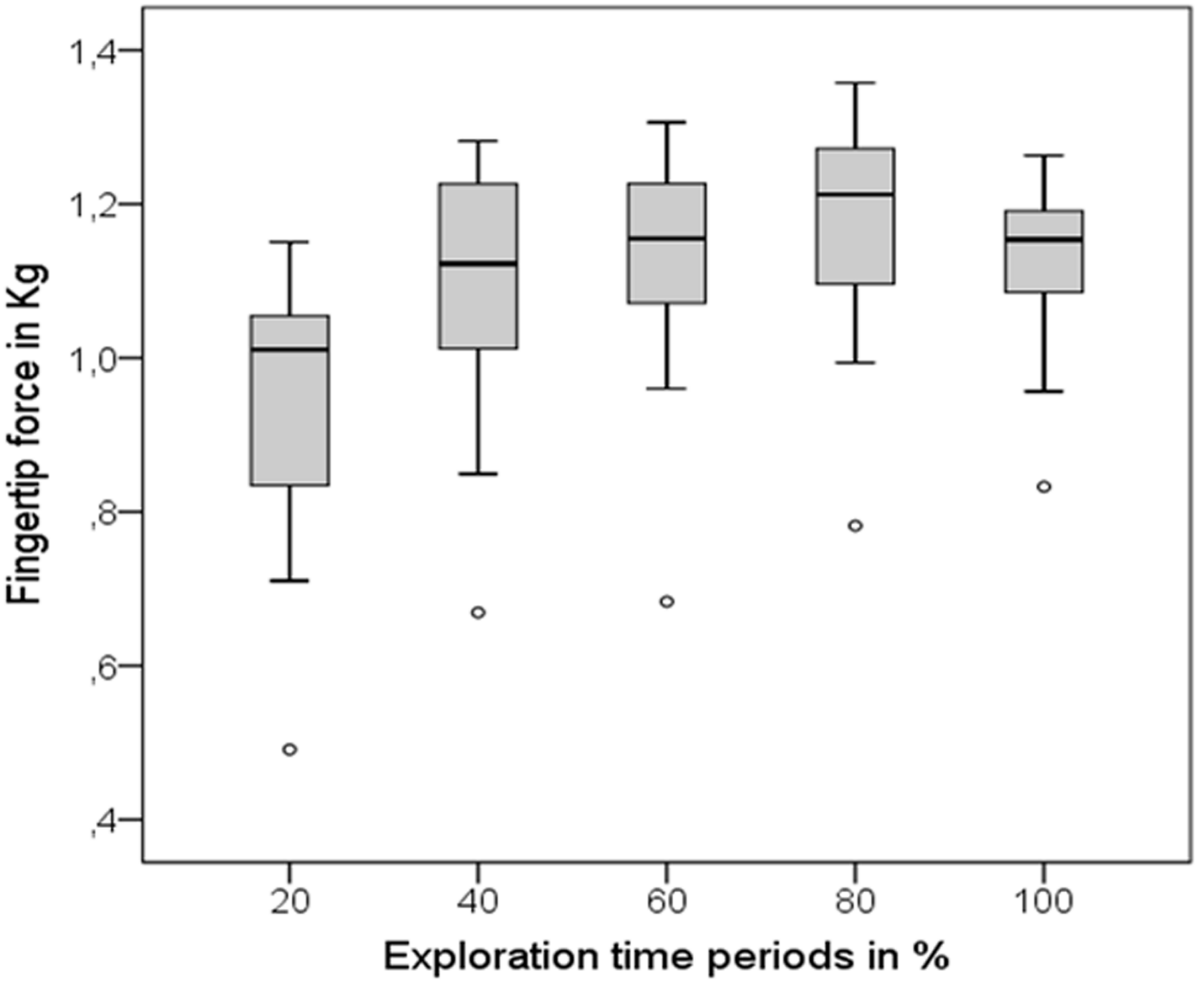
Level means and standard deviation of all 5 exploration time periods. Means are calculated from all 13 test stimuli across all participants (*N* = 20). Small dots depict outlier values.

A repeated measures one-way ANOVA demonstrated a significant difference between the five level means of Experiment A, *F*(3, 797) = 36.37, *p* < .0001 (Greenhouse-Geisser, Fig 5). This represented a medium effect (η^2^ = .124), showing that 12.4% of the variation in fingertip force was accounted for by the exploration time periods.

Post hoc comparisons applying Bonferroni correction showed significant differences between the first 20% of exploration time and all other exploration time periods. Additionally, the means of periods 40% and 80% were significantly different (Table 8). These results suggest that the biggest increase in fingertip pressure occurred systematically at the beginning (within the first 40 percent) of exploration time and remained high after that.

**Table 8:** Post Hoc: Bonferroni pairwise comparisons of all exploration time periods.

Exploration time periods		Mean difference	95% CI		*p*
			Lower level	Upper level	
20	40	-.172	-.234	-.111	.000
	60	-.212	-.288	-.137	.000
	80	-.254	-.334	-.175	.000
	100	-.220	-.301	-.139	.000
40	60	-.040	-.095	.014	.382
	80	-.082	-.147	-.018	.004
	100	-.048	-.120	.025	.637
60	80	-.042	-.094	.011	.244
	100	-.007	-.070	.055	1.000
80	100	.035	-.021	.090	.779

*Note*. Mean differences are based on estimated marginal means. Bonferroni corrected.

**Fig 6:**
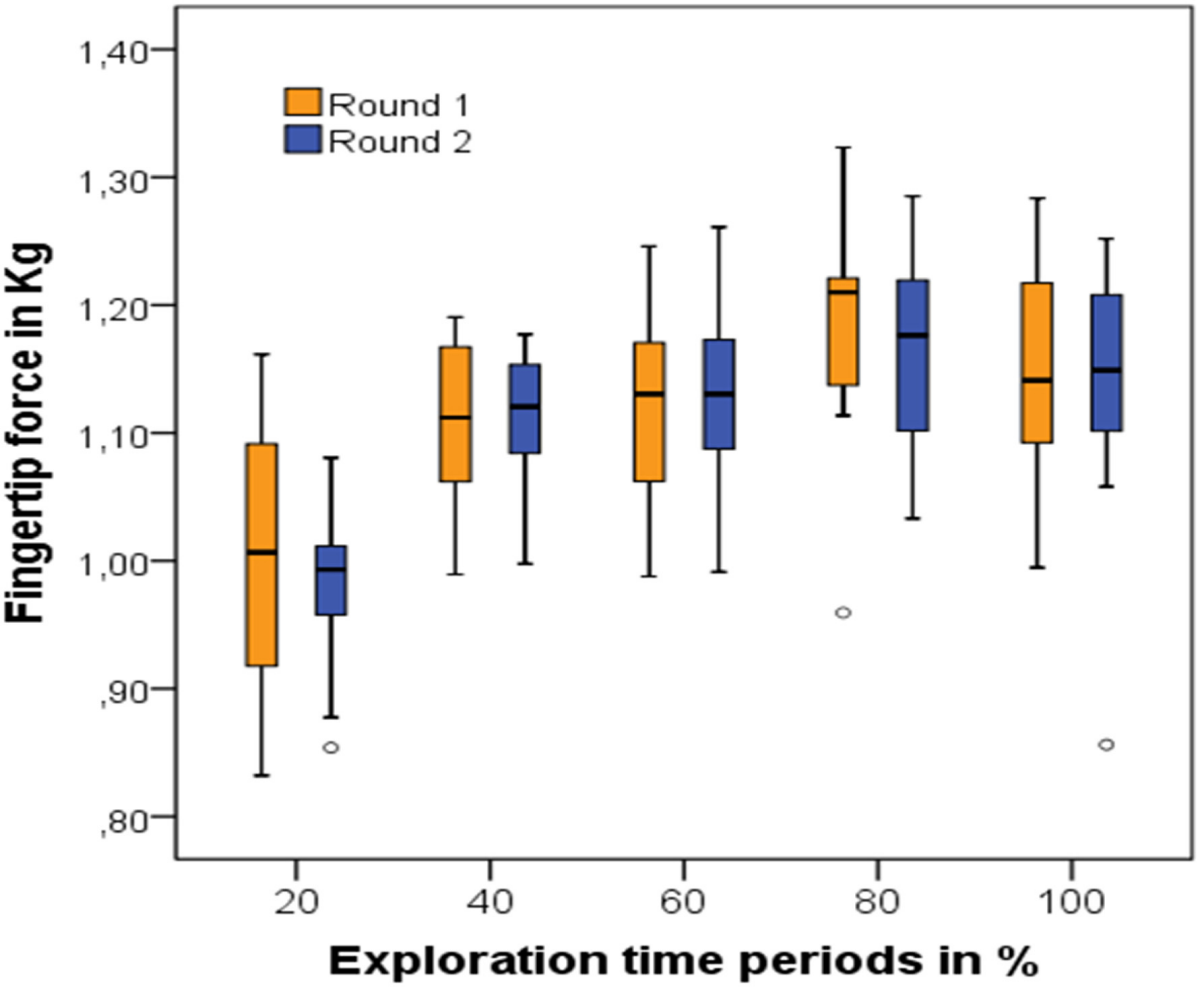
Level means and standard deviation of all 5 exploration time periods during both rounds of random stimulus presentation. Means are calculated from all 13 test stimuli across all participants (*N* = 19). Small dots depict outlier values.

The repeated measures one-way ANOVA of the data from Experiment B also demonstrated a significant difference between the five level means of both test rounds (Round 1: *F*(3,828) = 16.45, *p* < .0001; Round 2: *F*(3,822) = 22.01, *p* < .0001; Greenhouse-Geisser, Fig 6). This represented small to medium effects (Round 1: η^2^ = .063; Round 2: η^2^ = .082), with 6.3% and 8.2% variation in fingertip force explained by the exploration time periods.

Post hoc comparisons applying Bonferroni correction showed significant differences between the first 20% of exploration time and all other exploration time periods for both rounds. Additionally, the means of periods 40% and 60% differed from the 80% period during round 1 (Table 9). These results again indicate that the biggest increase in fingertip pressure occurred systematically at the beginning of exploration time and remained high after that.

**Table 9:** Post Hoc: Bonferroni pairwise comparisons of all exploration time periods.

		Round 1				Round 2			
Exploration time periods		Mean difference	95% CI		*p*	Mean difference	95% CI		*p*
			Lower level	Upper level			Lower level	Upper level	
20	40	-.107	-.172	-.041	.000	-.134	-.183	-.084	.000
	60	-.113	-.195	-.031	.001	-.145	-.212	-.078	.000
	80	-.194	-.277	-.112	.000	-.187	-.255	-.119	.000
	100	-.147	-.228	-.066	.000	-.156	-.224	-.087	.000
40	60	-.006	-.068	.055	1.000	-.011	-.060	.039	1.000
	80	-.088	-.151	-.025	.001	-.053	-.110	.004	.087
	100	-.040	-.113	.033	1.000	-.022	-.088	.045	1.000
60	80	-.082	-.142	-.021	.002	-.042	-.100	.015	.363
	100	-.034	-.105	.037	1.000	-.011	-.082	.061	1.000
80	100	.048	-.014	.109	.288	.032	-.026	.090	1.000

*Note*. Mean differences are based on estimated marginal means. Bonferroni corrected.

#### Exploratory analysis of fingertip pressure in milliseconds

**Fig 7:**
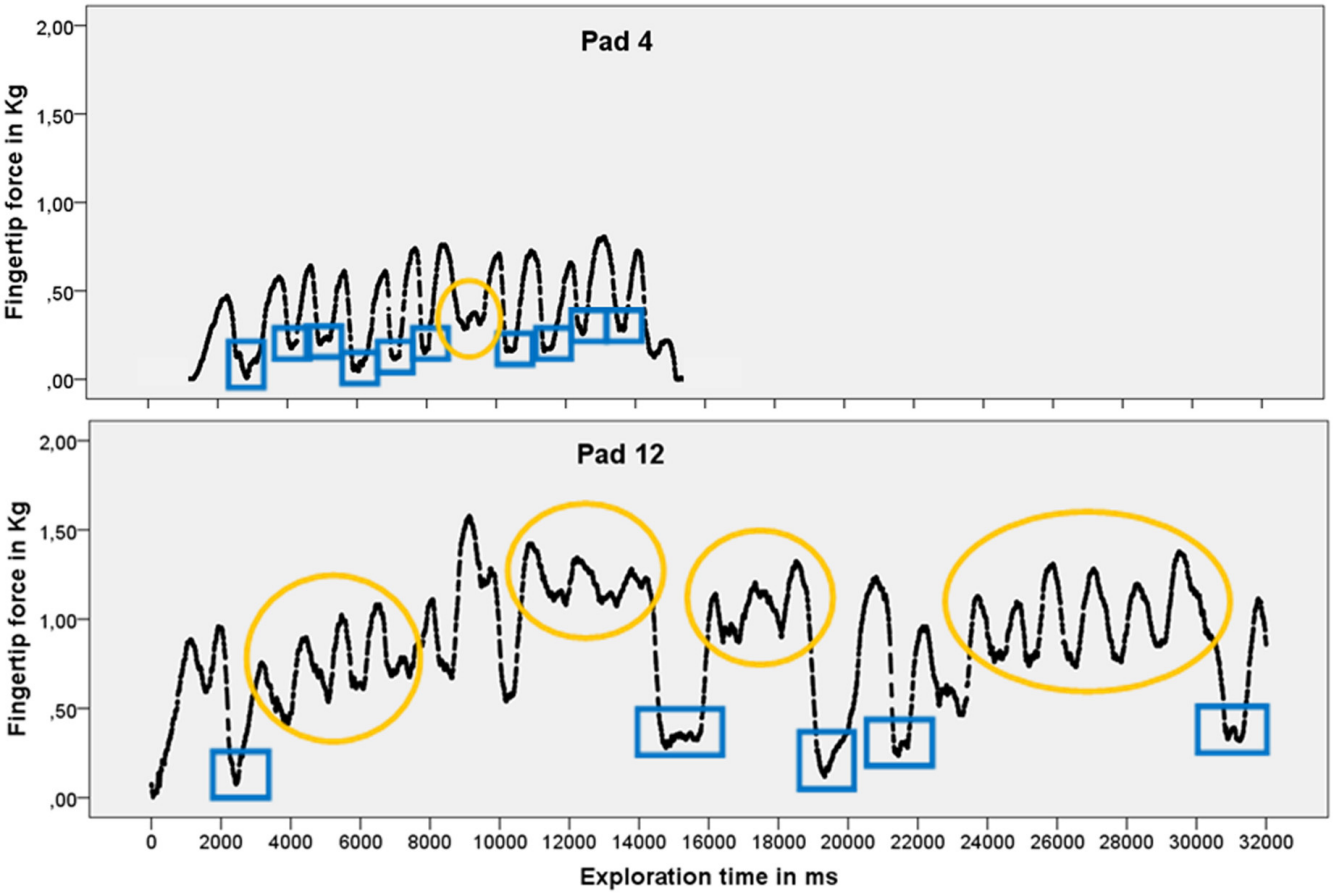
Temporal modulations of fingertip force of one participant. Time between data points is approximately 4 ms. Blue boxes indicate rotations. Yellow circles indicate exploration strategy 2 (up and down movements). All other peaks in fingertip force resulted from exploration strategy 1.

Exploratory analyses revealed that the fingertip force of each participant was under constant dynamic change during the exploration of each stimulus (see exemplary Fig 7 of a simple and a difficult stimulus). Similarly to the finger movements progressing up and down on the explored surface the applied pressure changed rhythmically.

Participants differed in their preferred exploration strategy but there were also many similarities. Most participants used a single finger at a time, with preference to the first and middle finger. Some participants occasionally switched to use the thumb or ring finger for a short period. There were predominantly two kinds of exploratory movements: 1) pulling the exploring finger orthogonally towards the body, lifting the finger and putting it back to the top to pull it towards the body again and again, resulting in the steady changes of exploratory pressure between peak and no pressure every few milliseconds; 2) quickly pushing and pulling the exploring finger back and forth across the surface causing pressure to increase steeply and to oscillate around an elevated plain.

With most participants the corresponding fingertip pressure was highest when the finger moved towards the body. However, a few participants preferred to use higher pressure when pushing the finger away from the body. Fingertip pressure was the lowest (almost zero) during rotation of the stimulus or when the fingers were detached.

Some participants used different exploration procedures for simple and difficult stimuli. For simple test stimuli they preferred the first exploration strategy. During the exploration of structures that were more difficult to detect an increase in back and forth movements occurred causing pressure to increase steeply and to oscillate around an elevated plain before dropping to zero for very short intervals while the finger was detached and the exploration procedure applied anew.

## Discussion

We found that mean fingertip force as well as temporal force modulations were strongly influenced by stimulus properties. While temporal modulations of the exploratory pressure were observed during the exploration of all stimuli, their distinct form depended on the stimulus’ detection difficulty. Overall, participants varied greatly in their preferred force [4,8,14,23]. But, by and large participants maintained their rank position: those who applied more fingertip force on simple stimuli also tended to use greater force for more difficult stimuli and vice versa. Similarly, other researchers reported stable relative force preferences across different settings and tasks within subjects [4,8,14,23].

The maximum force applied by participants lay between 1.40 kg (13.74N) and 4.99 kilograms (48.95N). These results are slightly higher but also more varied than the results reported by Keenan et al [29]. This difference may stem from the fact that the participants in the study by Keenan et al [29] did not have to accomplish a task other than applying as much pressure as they could. Both study samples had the same age range.

In our study, two participants used remarkably high fingertip forces from the start and reached mean forces of more than 2 kilograms. Many disorders influence haptic perception. While these changes may not be noticeable during daily activities they are measurable with rigorous tests. We have made an effort to exclude all test subjects with any kind of disorder or disease that may result in polyneuropathy or impaired circulation of the hands. A similar effort should have been made to exclude participants with psychological issues. Previous studies using difficult haptic relief stimuli have reported significant shortcomings in the perceptual capabilities of patients with poor insight like obsessive compulsive disorder [32] and anorexia nervosa [33–35]. In the present study psychiatric disorders were assessed only via self-report. To further investigate the scope of natural variation in fingertip forces used for exploration future studies should investigate whether subclinical manifestations of psychiatric disorders may have a significant impact on fingertip force and what systematics could be behind such effects.

Fingertip force and exploration time increased with increasing detection difficulty confirming Hypothesis 1 and 2 for both successive and random stimulus presentation.

As expected, exploration time increased with increasing detection difficulty. This effect was more pronounced during random stimulus presentation. The prominent drop in mean exploration time of test stimulus 13 in Experiment A should most likely be considered an expectation effect.

Test stimuli with higher detection difficulty were also explored with higher fingertip force than simpler ones. Furthermore, larger variations in force occurred with more difficult stimuli. We also observed a small but highly significant quadratic effect for stimuli with higher detection difficulty, indicating that the average applied force remained stable across the exploration of the more difficult stimuli. We hypothesized that participants would not increase their fingertip force further after they reached their personal threshold. Therefore, we expected the mean fingertip force to level off, possibly even drop, around test stimulus 10.

The mean haptic thresholds of the present sample were *M* = 9.65 (Exp. A) and *M* = 8.47 (Exp. B). They were similar to those of other young samples [30]. Fingertip force and exploration time reached their maximum between test stimuli 10 and 11 in both experiments. Consequently, participants seemed to reduce their efforts after sensory input ceased even during random stimulus presentation. This behaviour should be investigated further in future studies. All participants appeared alert and interested during the study. However, all participants were psychology students receiving credit points as their only reward. Therefore, intrinsic motivation to invest extra time and energy might have not been very high [36]. Alternatively, the effect may represent a true ceiling effect of perception. Mean pressure on the reference stimulus was much lower than the mean fingertip force used for the exploration of test stimulus 1 in all groups. Future researchers should consider using more stimuli with lower detection difficulty to investigate the scope of this effect.

As assumed under Hypothesis 3 we found an increase in fingertip force across the exploration time of each stimulus. We found a significant difference in fingertip force between the first and all four other exploration time periods. Additionally, there was a significant increase between the second and forth exploration time periods during successive presentation and from the second and third period to the forth during round 1 of experiment B (random stimulus presentation). These results suggest that the biggest increase in fingertip pressure occurred systematically at the beginning (within the first 40 percent) of exploration time and remained high after that. Since fingertip force oscillated approximately once every second and all zero-pressure values during initial finger-attachment and final finger-detachment were excluded, this effect is not merely due to the initial increase in fingertip force during the first contact. While individuals differ strongly in their preferentially applied force, the executed force was adapted to task properties from the very beginning of exploration. That means that all critical information is received during the initial contact phase and is directly transformed into the exploration procedure and force application.

**Fig 8:**
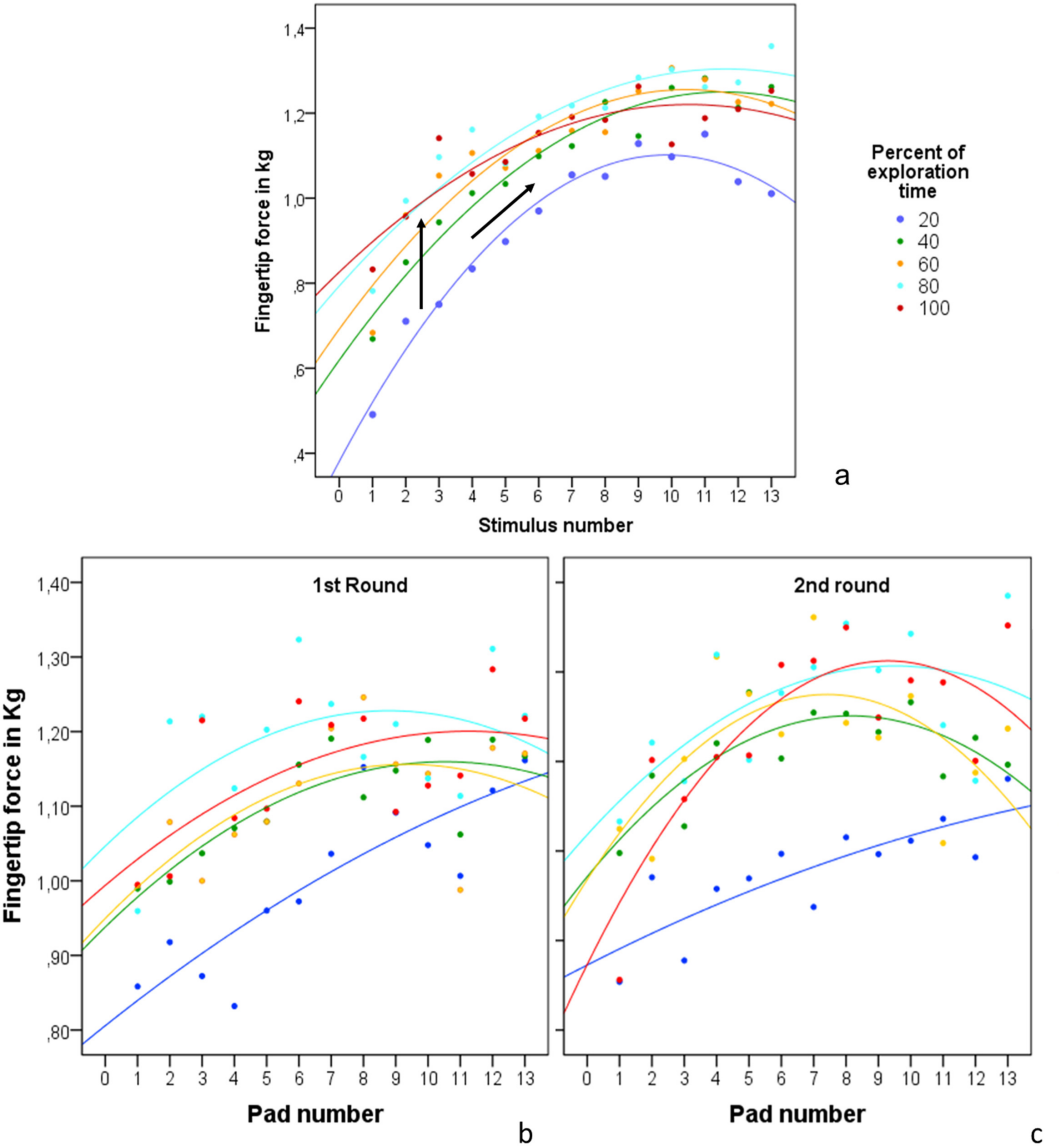
Mean fingertip pressure of each time period for all test stimuli. Fingertip force increased with increasing exploration time (vertical arrow) as well as with increasing detection difficulty (diagonal arrow). a) Successive stimulus presentation. b/c) Random stimulus presentation.

The strong variance in fingertip force during all 5 exploration time periods may be explained by differences in mean pressure between the different stimuli. As discussed under Hypothesis 1 fingertip forces were predicted by detection difficulty. With respect to the 5 exploration time periods this was especially evident at the beginning of exploration time (20% period) (Fig 8, diagonal arrow; For better visualization Fig 8 depicts only the respective means; distribution of the data may be seen in Tables 6 and 7). While during successive stimulus presentation the increase in fingertip force appears very orderly and may at least partially be due to an expectation effect of the participants: since stimuli presentation was in ascending order they might have intuitively increased their exploratory pressure with each new stimulus. However, the same effect occurred during random stimulus presentation (Fig 8c and 8d). Even though participants could not have known how difficult the next stimulus would be the linear increase of fingertip force with detection difficulty was evident after a few seconds of exploration time. Therefore, adaptation of exploratory force is not only very precise but also very fast.

While hardly comparable, it is odd to observe that the progression of the rise in fingertip force is similar across increasing detection difficulty and within the test stimulus exploration time. Detection difficulty delineates its typical change in mean pressure application in all 5 exploration time periods. Participants used less pressure to explore an easier stimulus than a difficult one both after 20% and after 80% of exploration time had passed (Fig 8). Independently of the initial pressure, fingertip force increased during the exploration of all stimuli and reached its peak after 80% of exploration time (Fig 8, vertical arrow). During the last fifth of exploration time mean pressure decreased again (not significantly). Whether mean pressure dropped during the 100% period due to exploration strategies or if the effect is due to a measurement error should be investigated in future studies. Even though we excluded all measurement values below 4 grams to exclude the attachment and detachment phases from the analysis, it is possible that the detachment of the finger from the test stimulus (drop in pressure) may have systematically skewed the results.

Furthermore, exploratory analyses of our high temporal resolution data revealed strong dynamic changes in the pressure application during test stimulus exploration with differences in the force dynamics of simple and difficult ones. We were able to detect two predominant exploration strategies after careful video analysis of the exploration process. Both strategies were used by all participants, but with varying frequency. For simple test stimuli most participants preferred to repeatedly stroke one or two fingers across the surface from top to bottom. The resulting exploratory pressure changed steadily between peak and low pressure every few milliseconds. During the exploration of structures that were more difficult to detect the second exploration strategy became more frequent. Fingertip pressure increased steeply and oscillated around an elevated plain while participants rubbed their fingertips back and forth across the surface. Katz (1925) observed that participants who were exploring different kinds of paper used stronger pressure when they were moving their finger towards the body than away from the body [37]. Our video analyses revealed a similar preference in most participants. A minority of participants, however, differed from the norm and used more pressure while pushing their fingertips away from the body.

Future studies should assess whether modulations of fingertip pressure reflect a neurophysiological process such as to maximise the function of the responding receptors when perception is liminal. In visual research for example it is well established that the focus during low light lies contiguous to the fovea, due to the absence of rod cells in the centre of the macula [38,39].

We found both fluctuations in force and moments of thorough absence of pressure during the exploration of all test stimuli. Participants rhythmically alternated between exploration periods and detaching their fingers from the surface. They detached their fingers to choose a new starting point, switch fingers or to rotate the stimulus. Again, it was Katz who pointed out before that during object exploration the surface is touched with interruptions rather than continuously [37]. Participants may use these techniques to reset mechanoreceptor adaptation. Extensive research conducted on single mechanoreceptors and their respective neurons has shown that continuous stimulation leads to a gradual decrease in receptor response [31]. Pressure fluctuations and breaks may interrupt this effect. Future analyses should assess the systematics of these temporal high-resolution data. Which primary force frequencies exist? Are they different for different materials? Does the length of the plateaus change systematically across exploration time?

## Conclusion

In the present study we attempted to glimpse into the dynamics of exploratory force modulations. We found that participants increase their exploratory force with increasing detection difficulty both during random and successive stimulus presentation. Participants used predominantly two exploration strategies. Their respective frequency changed with stimulus difficulty. We conclude that participants adapt their exploration characteristics in accordance with stimulus properties in order to improve their task performance (cf. [4,10–12]). These adaptations seem to be fine-tuned to even small changes in stimulus properties. Our results shed new light on the details of haptic exploration procedures.
